# Dynamic Diversity of NLR Genes in *Triticum* and Mining of Promising NLR Alleles for Disease Resistance

**DOI:** 10.3390/cimb43020069

**Published:** 2021-08-17

**Authors:** Xiaolong Li, Shifeng Cheng

**Affiliations:** Shenzhen Branch, Guangdong Laboratory of Lingnan Modern Agriculture, Genome Analysis Laboratory of the Ministry of Agriculture and Rural Affairs, Agricultural Genomics Institute at Shenzhen, Chinese Academy of Agricultural Sciences, Shenzhen 518120, China; lixiaolong@caas.cn

**Keywords:** NLRs, disease resistance, dynamic diversity, selective sweeps, transcriptome

## Abstract

Bread wheat is an essential crop with the second-highest global production after maize. Currently, wheat diseases are a serious threat to wheat production. Therefore, efficient breeding for disease resistance is extremely urgent in modern wheat. Here, we identified 2012 NLR genes from hexaploid wheat, and Ks values of paired syntenic NLRs showed a significant peak at 3.1–6.3 MYA, which exactly coincided with the first hybridization event between A and B genome lineages at ~5.5 MYA. We provided a landscape of dynamic diversity of NLRs from *Triticum* and *Aegilops* and found that NLR genes have higher diversity in wild progenitors and relatives. Further, most NLRs had opposite diversity patterns between genic and 2 Kb-promoter regions, which might respectively link sub/neofunctionalization and loss of duplicated NLR genes. Additionally, we identified an alien introgression of chromosome 4A in tetraploid emmer wheat, which was similar to that in hexaploid wheat. Transcriptome data from four experiments of wheat disease resistance helped to profile the expression pattern of NLR genes and identified promising NLRs involved in broad-spectrum disease resistance. Our study provided insights into the diversity evolution of NLR genes and identified beneficial NLRs to deploy into modern wheat in future wheat disease-resistance breeding.

## 1. Introduction

Infectious diseases are the main challenge in agricultural production and lead to heavy yield losses of many crops [1,2]. Bread wheat (*Triticum aestivum*) is one of the top three global food security crops, providing ~20% of calories and protein in modern human diets [3]. Global demand for wheat production has increased along with human population growth and is projected to require a yield increase of ~60% by 2050 [4,5]. Currently, bread wheat production is seriously threatened by varied diseases, such as *Fusarium* head blight (Fhb), powdery mildew, rust, and root rot, which are projected to lead to 20–30% yield loss, even up to 50% in wheat every year [6,7].

Nucleotide-binding leucine-rich repeat (NLR) proteins, encoded by one of the most variable gene families in plants, are intracellular immune receptors involved in disease resistance through their recognition of pathogen proteins [8]. For most plants, the N terminus of the NLR protein contains a Toll/interleukin-1 receptor (TIR) or a coiled-coil (CC) domain along with a central nucleotide-binding (NB) domain [9,10]. Plant NLR proteins can indirectly detect pathogen effectors by monitoring how they modify host targets. NLR proteins can also directly detect pathogen effectors through interactions between effectors and NLR domains or integrated domains [11]. The first NLR gene identified, *Hm1*, was cloned from maize (*Zea mays*) over 29 years ago [12]. Since then, many highly variable NLR gene loci that provide resistance to a variety of pathogens have been identified and isolated [11]. The release of high-throughput sequencing data enables genome-wide identification and analysis of NLR proteins [13,14] as well species-wide inventories of these proteins [8,15]. Release of the wheat reference genome (IWGSC RefSeq v1.0) [16] and mass resequencing data [17,18,19,20] have allowed us to identify the genome-wide repertoire of NLRs in wheat, determine their diversity in wheat relatives, and recover the diverse NLR gene pool through breeding and improvement of disease resistance in wheat.

Here, we identified genome-wide NLR genes and investigated the diversity of NLRs within/between *Triticum* and *Aegilops* based on whole-genome sequencing data. NLR genes in selective sweeps during domestication and improvement were further captured, and transcriptome data was used to identify promising functional NLRs for multiple disease resistance. This study lays a foundation for investigations of NLR diversity at the genus level in *Triticum* and *Aegilops* and will provide important gene resources of disease resistance for future wheat resistance breeding and improvement.

## 2. Materials and Methods

### 2.1. Identification of NLR Genes in Wheat

First, all protein sequences (FASTA file) and information about genomic positions (GFF file) of the hexaploid wheat reference genome “Chinese Spring” (IWGSC RefSeq v1.1) were downloaded from Unité de Recherche Génomique Info (URGI, https://urgi.versailles.inra.fr/download/iwgsc/IWGSC_RefSeq_Annotations/v1.1/, (accessed on 10 June 2021)). To get the NLR genes of wheat, the strategy of HMMsearch (hidden Markov model) with the NB-ARC domain HMM profile (PF00931) was carried out. We obtained the seed alignment file for the NB-ARC domain from the Pfam database (http://pfam.xfam.org, (accessed on 18 June 2021)) [21] and built an HMM file based on the obtained seed file using HMMER version 3.3.2 (http://www.hmmer.org, (accessed on 18 June 2021)) [22]. Next, we used the HMMsearch function of HMMER to search the annotated protein database of “Chinese Spring”. Genes on scaffolds were removed from subsequent analyses, and 2012 reliable NLR proteins were ultimately retained.

### 2.2. Chromosome Locations and Distribution of NLR Gene Clusters

The genomic distribution of identified wheat NLR proteins was displayed using TGT (Triticeae-Gene Tribe, http://wheat.cau.edu.cn/TGT/, (accessed on 20 June 2021)) [23]. We then split the wheat genome into 200 Kb slide windows using a Python script created in-house and counted the number of NLRs in each 200 Kb window using the BEDtools software (https://bedtools.readthedocs.io/en/latest/, (accessed on 20 June 2021)). We defined a window with at least two NLRs as the distribution of gene clusters. The genomic distribution of 200 Kb windows with different numbers of NLR proteins in 21 chromosomes was displayed using R package “RIdeogram” [24].

### 2.3. Identification of NLRs Synteny and Ka/Ks Evaluation

We extracted the syntenic wheat NLR pairs based on the high confidence syntenic triads published by Cristobal’s group and further verified them using TGT. The Ka (number of substitutions per nonsynonymous site) and Ks (number of substitutions per synonymous site) values of each NLR pair were calculated using TBtools (https://github.com/CJ-Chen/TBtools, (accessed on 20 June 2021)) [25]. The divergence time based on Ks was calculated as T = (2 × 6.1 ×10^−9^) × 10^−6^ [26].

### 2.4. Evaluation of Dynamic Diversity of NLR Genes in Triticum and Aegilops

The genotyping data of *Triticum* and *Aegilops* populations was downloaded from the Genome Variation Map (https://bigd.big.ac.cn/gvm, (accessed on 1 June 2021)) published by Lu’s group [18]. The number of SNPs in each group was counted using the VCFtools software (version 0.1.16) (https://github.com/vcftools/vcftools, (accessed on 1 June 2021)). We defined the upstream 2 Kb sequence of a gene as the promoter region and counted the number of SNPs in the genic and promoter regions of each NLR gene using BEDtools (v2.30.0) (https://bedtools.readthedocs.io/en/latest/, (accessed on 1 June 2021)). The occurrence rate of SNPs (ORS) was calculated using the number of SNPs on each NLR gene or its promoter region divided by number of SNPs on the chromosome where the gene is located.

### 2.5. Detection of Selective Signal during Domestication and Improvement

Four comparisons were conducted to identify selective sweeps across the whole genome: wild einkorn vs. domesticated einkorn; wild emmer vs. domesticated emmer; domesticated emmer vs. durum; landrace vs. cultivar. The levels of nucleotide diversity (π) and genetic differentiation (*Fst*) between groups were quantified in 50 Kb sliding windows with 5 Kb steps using the VCFtools software (version 0.1.16) (https://github.com/vcftools/vcftools, (accessed on 1 June 2021)). The π ratios were calculated using π_wild_/π_domesticated_ (domestication process) or π_domesticated_/π_cultivar_ (improvement process). The windows with the top 5% of π ratios and top 5% of *Fst* values were considered simultaneously as candidate windows under strong selective sweeps. All candidate windows were assigned to corresponding genes using BEDtools v2.30.0 (https://bedtools.readthedocs.io/en/latest/, (accessed on 1 June 2021)).

### 2.6. Transcriptome Data from the Pathogen Inoculation Experiment

The transcriptome data from the disease resistance experiment was downloaded from WheatOmics (http://202.194.139.32/expression/wheat.html, accessed on 28 June 2021) using the “requests” module in Python. All heatmap figures were generated using the “pheatmap” package (https://cran.r-project.org/web/packages/pheatmap/, (accessed on 30 June 2021)) in the R environment (https://www.r-project.org, (accessed on 30 June 2021)). The “scale = row” option was used to standardize the transcripts per million (TPM) values, and “cluster_row = TRUE” was used to perform gene clustering.

For the *Fusarium graminearum* inoculation experiment, the spikelet and rachis tissue inoculation with *F. graminearum*, DON, or water from the wheat resistant line (Fhb1+) and susceptible line (Fhb1−) was used for this study. In *F. graminearum*-inoculated samples, spikes were inoculated with *F. graminearum*, and the inoculated spikelet and corresponding rachis were sampled at 96 h after inoculation (hai). In the DON- and water-inoculated samples, spikes were inoculated with DON and water, and the inoculated spikelets were sampled at 12 hai. Three biological replications were carried out for each experiment [27]. RNA was extracted using the RNeasy Plant Mini Kit (QIAGEN, Valencia, CA, USA) from each replication of the experiments, and sequencing was performed using the Illumina HiSeq 2000 (Illumina, Inc., San Diego, CA, USA).

For the stripe rust and powdery mildew experiment, the wheat leaves inoculated with stripe rust or powdery mildew were collected at 0, 24, 48, and 72 hai. The experiment was carried out with three biological replications. Inoculated leaves were collected and frozen immediately in liquid nitrogen. The cDNA libraries were constructed by PCR amplification and sequenced using the Illumina HiSeq™ 2000 platform. The expression level of each gene model in each sample was measured using the reads per kilobase of exon model per million of aligned reads (RPKM) values [28].

For the *Xanthomonas translucens* inoculation experiment, leaves of 49-day-old plants were inoculated using *Xanthomonas translucens* suspension in water and inoculated with water as controls. Leaves and root tissues were then harvested 1 day after inoculation. Three biological replications were carried out for each treatment, and each with pooled samples from two independent plants of each replication. Total RNA was isolated from leaves and roots inoculated with *Xanthomonas translucens*, and RNA-seq libraries were constructed using the MGX-Montpellier GenomiX platform. Sequencing was performed using an Illumina Hiseq 2500 (Illumina Inc., San Diego, CA, USA) [29].

## 3. Results

### 3.1. Genome-Wide Identification of NLR Proteins in Wheat

**Figure 1:**
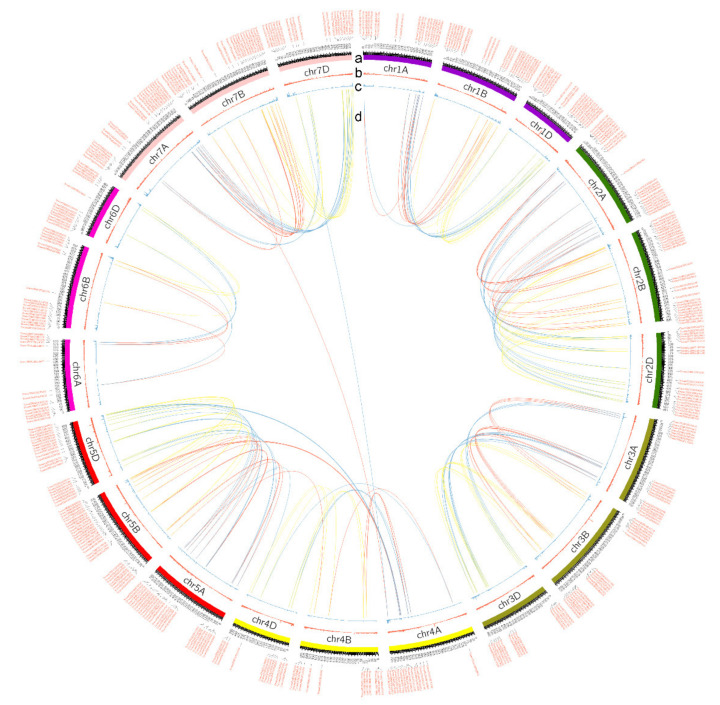
The summary of identified NLR proteins in wheat. (**a**) The Circos plot displays the genomic distribution of NLRs in 21 chromosomes; (**b**) the red bar represents the number of annotated high-confidence genes in each 200 Kb window and was plot using Circos; (**c**) the blue bar represents the number of NLRs in each 200 Kb window; (**d**) the synteny of paired NLR proteins on A, B, and D subgenomes. Red lines connect A and B subgenome; yellow lines connect B and D subgenome; blue lines connect A and D subgenome. This figure was plotted using Circos-0.69-9.

A total of 2012 high-confidence NLR proteins were identified in the hexaploid wheat reference genome “Chinese Spring” (IWGSC RefSeq v1.1) [16], and 625 (31.06%), 785 (39.02%), and 602 (29.92%) NLRs were identified in the A, B, and D subgenomes, respectively (Table S1). As noted in previous reports, NLR genes are generally divided into four classes based on their conserved domain: TIR-NLR, CC-NLR, CCR-NLR, and NB-LRR, which are canonical NLR domains TIR, CC, RPW8-like coiled-coil (CCR), and leucine-rich repeats (LRRs), respectively [8]. Details of the conserved domain and gene annotation of each NLR protein, which provide basic gene resources for subsequent analyses, are in Table S1. Based on the genomic position, we mapped each NLR to 21 chromosomes of the wheat genome and found that NLRs are frequently distributed on telomeres of each chromosome (Figure 1 and Figure S1). We also observed that NLRs were most enriched in chromosome 1B, accounting for 3.05% of total genes on 1B (Table S2), in which a large chromosome segment introgressed from rye (*Secale cereale* L.) has been reported [30,31]. The fewest NLRs were identified on chromosomes 4B (26 NLRs, 0.65%) and 4D (21 NLRs, 0.57%) (Table S2; Figure S2).

**Figure 2:**
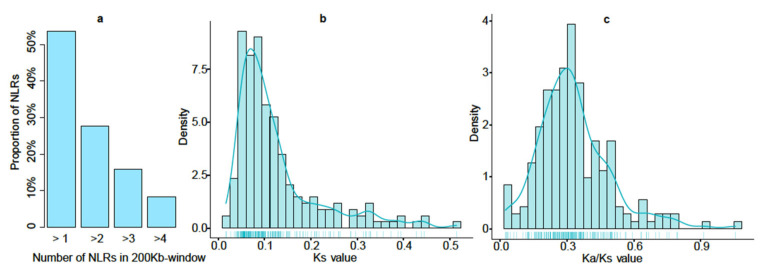
The distribution of NLRs in 200 Kb windows and the calculation of Ka (number of substitutions per nonsynonymous site) and Ks (number of substitutions per synonymous site) values of paired NLRs. (**a**) The proportion of NLR proteins distributed on each 200 Kb slide window as gene clusters; the distribution of Ks values (**b**) and Ka/Ks ratio (**c**) of paired NLRs.

Considering that NLRs are distributed in the genome as gene clusters [32], we further split the wheat genome into 200 Kb-based slide windows and counted the number of NLRs in each window. Results showed that 2012 NLRs were distributed in 1389 200 Kb windows and 407 of these windows had at least two NLR proteins (accounting for 53.58% of total NLRs); 147 had at least three NLRs (accounting for 27.73% of total NLRs); 68 had at least four NLRs (accounting for 15.91% of total NLRs); and 29 had at least five NLRs (accounting for 8.20% of total NLRs) (Figure 2a, Figure S3 and Table S3). This is consistent with the observation from Arabidopsis, in which an average of 59% of NLRs are distributed in the genome as clusters [8].

Further, we identified syntenic wheat NLR pairs based on the high-confidence syntenic triads published by Cristobal’s group [33]. A total of 200 NLR pairs (Figure 1; Table S4) and 91 1:1:1 (on A, B, and D subgenomes) high-confidence syntenic NLR triads were identified among A, B, and D subgenomes (Table S5). We also calculated the Ka (number of substitutions per nonsynonymous site) and Ks (number of substitutions per synonymous site) values of each NLR pair using TBtools (https://github.com/CJ-Chen/TBtools, (accessed on 20 June 2021)) [25]. The distribution of Ks values showed a significant peak at 0.04–0.08 (Figure 2b; Table S4), indicating about 3.1–6.3 MYA (million years ago) calculated using [26]:

T = Ks ÷ (2 × 6.1 ×10^−9^) × 10^−6^.

This coincides with the first hybridization event between A and B genome lineages that occurred ~5.5 MYA, which directly led to the origin of the D genome lineage [34]. Moreover, the Ka/Ks ratios of all NLR pairs are less than 1 (Figure 2c; Table S4), suggesting that many deleterious mutations have occurred and these NLR proteins are undergoing purifying selection [35].

### 3.2. Dynamic Diversity of NLR Proteins in Evolution of Triticum and Aegilops

The release of resequencing data at the genus level of wheat allows us to survey the dynamic diversity of key genes associated with important agronomic traits during domestication and improvement. In this study, we downloaded genotyping data of *Triticum* and *Aegilops* populations from the Genome Variation Map (https://bigd.big.ac.cn/gvm, accessed on (1 June 2021)) published by Lu’s group [18]. The genotypes of 261 wheat accessions and wheat relatives were selected for subsequent analysis (Table S6). In these accessions, a total of four lineages were included: AA lineage (*T. monococcum*/*T. urartu*, 91 accessions), AABB lineage (*T. turgidum*, 70 accessions), AABBDD lineage (*T. aestivum*, 70 accessions), and DD lineage (*Ae. tauschii*, 30 accessions). These lineages were further assigned to nine groups: wild einkorn (31, AA), domesticated einkorn (31, AA), urartu (29, AA), wild emmer (28, AABB), domesticated emmer (29, AABB), durum (13, AABB), landrace (45, AABBDD), cultivar (25, AABBDD), and diploid DD (30, DD, including strangulata, meyeri, and anathera) (Table S6) [18].

**Figure 3:**
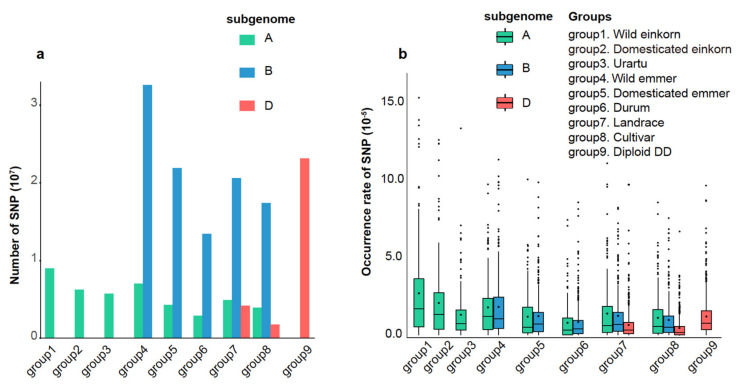
The total number of SNPs in each group and occurrence rate of SNP on NLRs for nine groups in different subgenomes. (**a**) The total number of SNPs in each group. Green bars represent the A subgenome; blue bars represent the B subgenome; red bars represent the D subgenome; (**b**) the occurrence rate of SNP on NLRs for nine groups in different subgenomes. Green boxes represent the A subgenome; blue boxes represent the B subgenome; red boxes represent the D subgenome. The rhombic point in each bar represents the average value. Occurrence rate = number of SNPs on each NLR gene/number of SNPs on the chromosome in which the gene is located.

Based on the genotyping data, we evaluated the diversity of each of the nine groups using single nucleotide polymorphism (SNP) counts in each subgenome (Table S7). Results showed that wild einkorn and wild emmer had higher numbers of SNPs in the A genome lineage, and tetraploid wild emmer and domesticated emmer had higher numbers of SNPs in the B genome lineage (Figure 3a). For the D genome lineage, the diploid DD group consisting of subspecies strangulata and varieties meyeri and anathera had many more SNPs than the D genome from hexaploid wheat (Figure 3a). These findings suggest that wild progenitors and wheat relatives have maintained higher diversity, which might have been lost during polyploidization (only a few accessions of *Ae. tauschii* contributed to the origin of hexaploid wheat) and domestication of wheat [36,37]. Investigating the diversity patterns of NLRs based on the genotyping data of *Triticum* and *Aegilops* populations is thus beneficial for the discovery of new elite NLR genes to improve the resistance of wheat.

**Figure 4:**
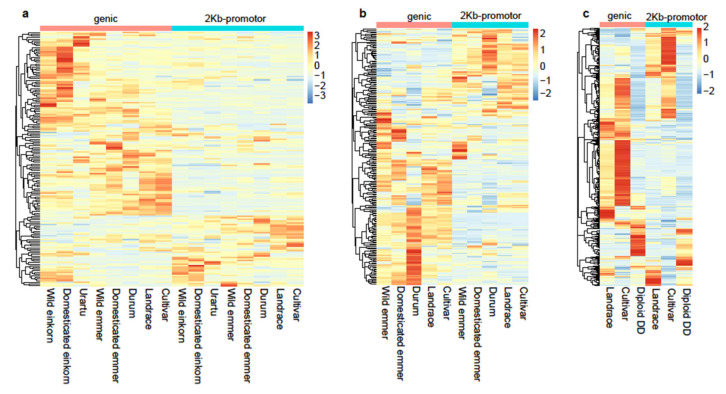
The heatmap of ORS (occurrence rate of SNP) on genic regions and 2 Kb-promoter regions of all NLRs with SNPs for nine groups in different subgenomes. (**a**) The heatmap for A genome lineages in wild einkorn, domesticated einkorn, urartu, wild emmer, domesticated emmer, durum, landrace, and cultivar groups; (**b**) the heatmap for B genome lineages in wild emmer, domesticated emmer, durum, landrace, and cultivar groups; (**c**) the heatmap for D genome lineages in landrace, cultivar, and diploid DD groups. Red cells represent the higher ORS, and blue cells represent the lower ORS.

To investigate the diversity variation of NLRs from different lineages in the nine groups, we calculated the occurrence rate of SNP (ORS = number of SNPs on each NLR gene/number of SNPs on the chromosome where the gene is located) of each NLR gene (Table S8) and its 2 Kb-promotor region (Table S9). Fewer NLR genes lacking SNPs (27.67% in genic region and 38.17% in promoter region of NLRs) were observed for the D genome lineage (Table S10; Figure S4), suggesting that NLRs are more conserved in A and B genome lineages than in D genome lineages. In D genome lineages, a significantly high proportion (12.44%) of NLRs with SNPs occurred only in the Diploid DD group, indicating that more unexplored diversity of NLRs was stored in diploid DD relatives (strangulata, meyeri, and anathera). Further, we calculated the ORS of each NLR with SNPs. As expected, the diversity of NLR genes has been maintained in wild progenitors and wheat relatives (Figure 3b). The heatmap showed that most NLRs with SNPs had an opposite pattern between the genic region and 2 Kb-promoter region in A and B genome lineages (Figure 4a,b), such as higher ORS in the genic region and lower ORS in the promoter region. In the D genome lineage, most NLRs with SNPs had an opposite pattern between the genic region and 2 Kb-promoter region only in landrace and cultivar groups; a similar pattern was observed in the diploid DD group (Figure 4c). This may be due to the evolution of different genome lineages, as well as sub/neofunctionalization and loss of duplicated genes, with SNPs in the genic region potentially contributing to the gene loss, while SNPs in the promoter region contributed to the differential gene expression [38].

### 3.3. NLRs Carried Selective Signals during Wheat Domestication and Improvement

In our analysis, studied accessions included wild einkorn, domesticated einkorn, wild emmer, domesticated emmer, durum, landrace, and cultivars, which allowed us to capture the genomic selective signals during domestication and improvement. To identify the selected wheat NLR genes, we scanned the whole genome to identify the genomic windows carrying selective signals using two thresholds to define the regions: (1) top 5% of π (nucleotide diversity) ratio, using wild type divided by domesticated type or domesticated type divided by improved type; and (2) top 5% of *Fst* value, an index of genetic differentiation between two groups.

**Figure 5:**
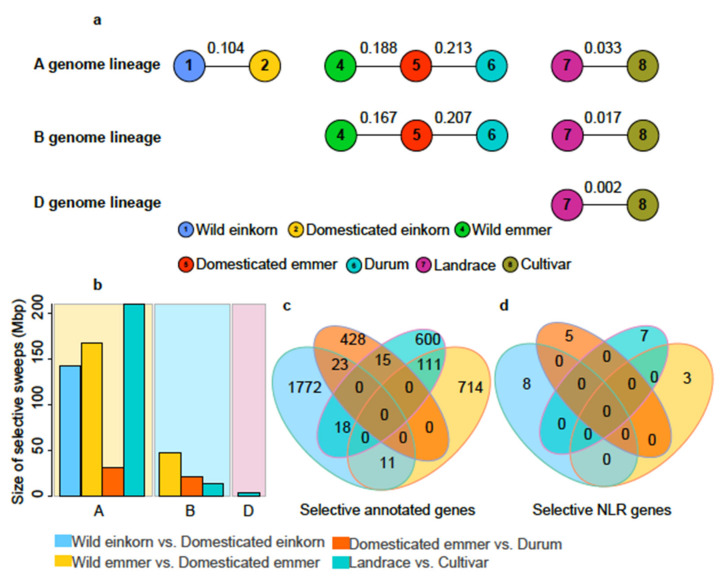
The genetic differentiation (*Fst*) between two groups and the identification of selective sweeps. (**a**) The evaluation of genetic differentiation between two groups using *Fst* values. Circles with number represent the different groups. The values on the lines represent the *Fst* values; (**b**) the size of selective sweeps in the A, B, and D genomes from four comparisons; (**c**) the overlapped annotated genes among four comparisons; (**d**) the overlapped NLR genes among four comparisons. Blue represents wild einkorn vs. domesticated einkorn; yellow represents eild emmer vs. domesticated emmer; red represents domesticated emmer vs. durum; green represents landrace vs. cultivar.

In this study, four comparisons were used to perform this analysis: wild einkorn vs. domesticated einkorn; wild emmer vs. domesticated emmer; domesticated emmer vs. durum; and landrace vs. cultivar. For *Fst*, the highest degree of differentiation was observed between domesticated emmer and durum for A (0.213) and B (0.207) genome lineages, while the lowest was observed between landrace and cultivar (A: 0.033; B: 0.017). For the three subgenomes, the A genome had the highest degree of differentiation, followed by the B genome, then the D genome (Figure 5a). As expected, the nucleotide diversity showed that wild and progenitor types have higher diversity and the B subgenome has the highest diversity in tetraploid and hexaploid wheat. In contrast, the D subgenome has relatively low diversity in hexaploid wheat (Figure S5), which had been reported in a previous study [18].

Further, a total of 142.97 Mbp, 215.21 Mbp (168.09 Mbp in the A genome and 47.12 Mbp in the B genome), 51.18 Mbp (30.65 Mbp in the A genome and 20.53 Mbp in the B genome), and 226.57 Mbp (210.30 Mbp in the A genome, 13.25 Mbp in the B genome, and 3.02 Mbp in the D genome) genomic windows were identified as candidate regions carrying selective signals from wild einkorn vs. domesticated einkorn, wild emmer vs. domesticated emmer, domesticated emmer vs. durum, and landrace vs. cultivar, respectively (Figure 5b, Figures S6 and S7; Table S11). A larger region had undergone selection after domestication (wild einkorn vs. domesticated einkorn, wild emmer vs. domesticated emmer) rather than after improvement (domesticated emmer vs. durum, and landrace vs. cultivar). We found that chromosome 4A contributed to 75.14% of selective sweeps of the A genome in the wild emmer vs. domesticated emmer comparison and 86.86% in the landrace vs. cultivar comparison (Table S11), which is due to the large introgression from wild emmer reported in previous studies [17,20].

In these candidate selective regions, a total of 1824, 836, 466, and 744 annotated genes were respectively identified from wild einkorn vs. domesticated einkorn, wild emmer vs. domesticated emmer, domesticated emmer vs. durum, and landrace vs. cultivar (Table S12), including eight, three, five, and seven NLR genes (Table S13). NLR genes were not significantly enriched in genomic regions carrying selective signals (*p* > 0.05). Additionally, we found fewer selected annotated genes shared in four comparisons (Figure 5c; Table S12) occurring, at best, in two comparisons, and each of the selected NLR genes were unique for each of the four comparisons (Figure 5d). Moreover, five selected NLR genes (*TraesCS3A02G095700*, *TraesCS3B02G111400*, *TraesCS5A02G069600*, *TraesCS7B02G024800*, and *TraesCS2B02G284500*) appeared in 1:1:1 high-confidence syntenic NLRs triads, and only genes *TraesCS3A02G095700* and *TraesCS3B02G111400* were paired (Table S5), suggesting a preference effect of genome selection.

### 3.4. Transcriptome Profiles of the Beneficial NLR Alleles for Disease Resistance

Transcriptome data from four experiments of pathogen inoculation were used to identify candidate functional NLRs for disease resistance in wheat, including *Fusarium* head blight (Fhb), powdery mildew, stripe rust, and *Xanthomonas translucens*. In the *Fusarium graminearum* inoculation experiment, Fhb-resistant (Fhb1+) and Fhb-susceptible (Fhb1−) wheat lines were separately inoculated with *F. graminearum* and deoxynivalenol (DON), and samples were inoculated with water as the control (https://www.ncbi.nlm.nih.gov/bioproject/PRJNA273659, (accessed on 28 June 2021)). The transcriptome data was downloaded and average values of three replications was used to plot the heatmap. We performed gene clustering using “cluster_row = TRUE” and the clustering tree showed four clades, suggesting four expression patterns from expressions of 2012 NLR genes (Figure S8A; Table S14). In pattern 1, NLR genes had a high expression level in Fhb1+ and Fhb1− wheat lines inoculated with water, while in pattern 2, NLR genes had a high expression level in Fhb1+ and Fhb1− lines inoculated with DON after 12 h. In pattern 3, NLR genes were only highly expressed in Fhb1− wheat lines inoculated with water after 12 h. In pattern 4, four expression cases were observed in the NLR genes: (1) high expression only in Fhb1+ lines with first-round *F. graminearum* inoculation after 96 h; (2) high expression only in Fhb1− lines with first-round *F. graminearum* inoculation after 96 h; (3) high expression only in Fhb1+ lines with second-round *F. graminearum* inoculation after 96 h; and (4) high expression only in Fhb1− lines with second-round *F. graminearum* inoculation after 96 h (Figure S8A). These results suggested that diverse defense response mechanisms of NLR genes have evolved for Fhb in Fhb1+ and Fhb1− wheat lines.

In the powdery mildew inoculation experiment, wheat lines were inoculated with powdery mildew for 24, 48, and 72 h, and noninoculation lines were used as a control [28]. A total of five expression patterns of NLR genes were observed (Figure S8B; Table S15). In pattern 1, NLR genes only had a high expression level after 24 h of inoculation with the powdery mildew pathogen, and NLR genes only had high expression after 48 h of inoculation with the powdery mildew pathogen in pattern 4. In pattern 2, NLR genes had a high expression level in the noninnoculation control and after 72 h of inoculation with the powdery mildew pathogen, while pattern 3 only had a high expression level in the noninnoculation control. In pattern 5, NLR genes (77.43%) were highly expressed after 48 and 72 h of inoculation, and higher expression levels occurred with longer inoculation time, suggesting the positive response of NLRs to the powdery mildew pathogen in wheat. Similarly, in the stripe rust inoculation experiment, most NLR genes had higher expressions after 24, 48, and 72 h of inoculation than in the noninoculation control (Figure S8C; Table S16).

In the *X. translucens* inoculation experiment, wheat leaf and root were inoculated with *X. translucens*, and water was used as a control (https://www.ncbi.nlm.nih.gov/bioproject/PRJEB21835/, (accessed on 28 June 2021)). As expected, the heatmap of expression showed that most NLR genes had high tissue-specific expression in the root (Figure S8D; Table S17), and compared with the control, a higher expression level was observed in wheat root inoculated with *X. translucens*. This suggests that *X. translucens* causes greater harm to the wheat root and NLR genes are involved in the positive response.

**Figure 6:**
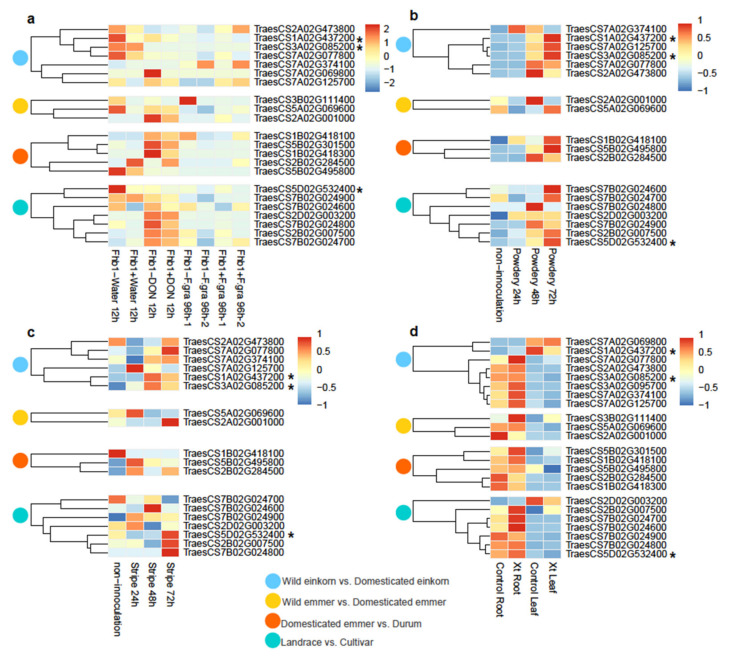
The expression heatmaps of selected NLR genes from four comparisons. Red cells indicate higher expression levels and blue cells represent lower expression levels. Blue circles represent wild einkorn vs. domesticated einkorn; yellow circles represent wild emmer vs. domesticated emmer; red circles represent domesticated emmer vs. durum; green circles represent landrace vs. cultivar. The asterisks beside the gene IDs represent the NLR genes involved in stripe rust resistance reported by Cristobal’s group (Ramírez-González et al., 2018). (**a**) The expression heatmap from the *Fusarium graminearum* infects wheat spikes and causes *Fusarium* head blight (FHB). Fhb1+ represents resistant lines and Fhb1− represents susceptible lines. −1 and −2 represent two replications; (**b**) the expression heatmap from the powdery mildew pathogen innoculation; (**c**) the expression heatmap from stripe rust pathogen innoculation; (**d**) the expression heatmap from *Xanthomonas translucens* (Xt) pathogen infection. Plants inoculated with water were used as controls. Data was downloaded from http://202.194.139.32/expression/wheat.html (accessed on 28 June 2021). This figure was generated using the R package “pheatmap”. Genes without expression data are not shown. The experiment was carried out with three biological replications, and average values of the three replications were used to plot the heatmap.

Additionally, we found that 195 of the 2012 identified NLR genes were reported to be associated with abiotic and/or disease stress [33], including two NLRs that responded to Fhb, 43 that responded to powdery mildew, and 118 that responded to stripe rust (Table S18). We also profiled the expression patterns of selected genes from four comparisons: wild einkorn vs. domesticated einkorn, wild emmer vs. domesticated emmer, domesticated emmer vs. durum, and landrace vs. cultivar (Figure 6). The expression patterns of selected NLR genes were similar to patterns of all annotated NLR genes, and no significant differences were observed for each selected NLR gene in four comparisons. Three selected NLR genes, *TraesCS1A02G437200*, *TraesCS3A02G085200*, and *TraesCS5D02G532400*, have been reported to be involved in stripe rust resistance by Cristobal’s group [33]. We also found that these three genes not only positively responded to stripe rust (Figure 6c) but also responded to powdery mildew (Figure 6b and Figure S9) yet had no significant response to Fhb (Figure 6a) or *X. translucens* (Figure 6d). Further, the dominant effect of genome selection for disease resistance was significant in the B genome lineage (Figure 6).

Our analysis indicates that NLR genes function directly in wheat disease resistance, and some have broad-spectrum resistance for multiple wheat diseases. The transcriptome profiles of NLR genes for different wheat diseases will provide guidance for future breeding and identified functional and beneficial NLR alleles can be used directly in the disease-resistance breeding of wheat.

## 4. Discussion

In this study, we identified the NLR gene atlas from the hexaploid wheat reference genome “Chinese Spring” (IWGSC RefSeq v1.1) and investigated the landscape of diversity of NLRs in *Triticum* and *Aegilops* populations. Consistent with previous studies [39,40], we found that wild progenitors and wheat relatives constitute reservoirs of genetic diversity of NLR genes, which can be exploited and deployed in modern wheat for disease resistance, especially for diploid DD relatives (strangulata, meyeri, and anathera). Furthermore, we quantified the genetic diversity of annotated NLR genes using ORS and constructed a map of dynamic diversity at the genus level for *Triticum* and *Aegilops*. This will provide important guidance for choosing NLR genes in future wheat disease-resistance breeding.

Based on the genotyping data of *Triticum* and *Aegilops* populations, we reanalyzed the allele frequency changes in NLR genes on A, B, and D genome lineages during domestication and improvement. Similar to previous studies [17,18], we found the A genome lineage has undergone the strongest selection in wheat evolution, and a large genomic region contributed to most allele frequency changes of 4A in tetraploid emmer and hexaploid wheat. This is consistent with previous reports showing that a large alien segment introgressed into 4A from wild emmer to hexaploid wheat, which also explains the maintenance of genetic diversity in the A and B genomes [20]. Two questions arise from these findings: (1) what sources have contributed to the diversity of tetraploid emmer? (2) How can the genomic effects of introgression and selection be distinguished? Collection of additional resources and methodological development are required to further explore these questions.

Transcriptome data from four pathogen inoculation experiments enabled us to profile the expression patterns of annotated NLRs in wheat. We verified three NLR genes reported to be associated with stripe rust resistance, and broad-spectrum resistance for multiple wheat diseases was observed. Moreover, these three NLR genes have also undergone strong selection in wheat evolution, and thus should be deployed into modern wheat as candidate-beneficial alleles to satisfy the broad-spectrum and effective long-term resistance goals of future resistance breeding and improvement of wheat.

A limitation of this study is that all NLR genes were identified from a single reference genome, which severely limited the discovery of new NLR alleles. How to fully capture the reservoir of NLR genes in the genera level is thus a focal issue for future studies. In Arabidopsis, resistance gene enrichment sequencing (RenSeq) analysis has been carried out for 64 accessions and a complete species-wide pan-NLRomes was constructed [8]. In wild diploid wheat, RenSeq was performed to fully retrieve disease-resistance genes from wild relatives [15]. Combined with associated genetic data, pan-genome variation was exploited to rapidly clone resistance genes. Considering the limitation of the single reference genome, more high-quality genomes and a pan-genome of wheat and its relatives will be required to reveal the global diversity. In 2020, the release of 10+ wheat genomes provided a landscape of global variation and a basis for the discovery of functional genes [41], but only for modern wheat cultivars. The genomes of wild progenitors and wheat relatives, especially for the pan-genome, are still required to identify new NLRs, such as diploid DD lineage strangulata, meyeri, and anathera. Song’s group has assembled four new reference genomes for the diploid DD lineage and established a rapid introgression platform for wheat breeding and improvement [42]. This will help to restore the diversity of the modern wheat D genome, which seriously restricts current selective breeding. Additionally, Kong’s group first provided a complete genome assembly of *Thinopyrum elongatum*, which was used for hybridization breeding of wheat as a distant species, and cloned the *Fhb7* gene involved in Fhb resistance [43]. Further, evidence supports that the *Fhb7* gene of *T. elongatum* was gained from an endophytic *Epichloe* species via horizontal gene transfer (HGT). It is likely that more of these events will be traced with the release of more genomes and the pan-genome of wheat progenitors and relatives.

## 5. Conclusions

In this study, we provide a repertoire of NLR genes from hexaploid wheat and a landscape of dynamic diversity of NLRs in *Triticum* and *Aegilops*. We found that NLR genes have higher genetic diversity in wheat progenitors and wild relatives. Further, selection analysis suggests that fewer NLR genes carry a selective signal, and each of the selected NLRs is unique. Moreover, we identified an alien introgression of chromosome 4A in tetraploid emmer, which was similar to that occurring in hexaploid wheat. We also profiled the expression pattern using transcriptome data from four experiments of disease resistance and identified promising NLR genes involved in the broad-spectrum resistance to multiple wheat diseases. Our study provides insights into the diversity evolution of NLR genes in wheat and identifies beneficial NLR alleles to deploy into modern wheat for future disease-resistance breeding.

## Data Availability

Not applicable.

## Supplementary Materials

The following are available online at https://www.mdpi.com/article/10.3390/cimb43020069/s1.
