# Supplementary material for: Dynamic Diversity of NLR Genes in Triticum and Mining of Promising NLR Alleles for Disease Resistance
Source: Curr Issues Mol Biol. 2021 Aug 17;43(2):965–77. doi: 10.3390/cimb43020069 (PMC8929112; doi:10.3390/cimb43020069)
Supplement: Supplementary file 1 [file cimb-43-00069-s001.zip › Figure S8.pdf]

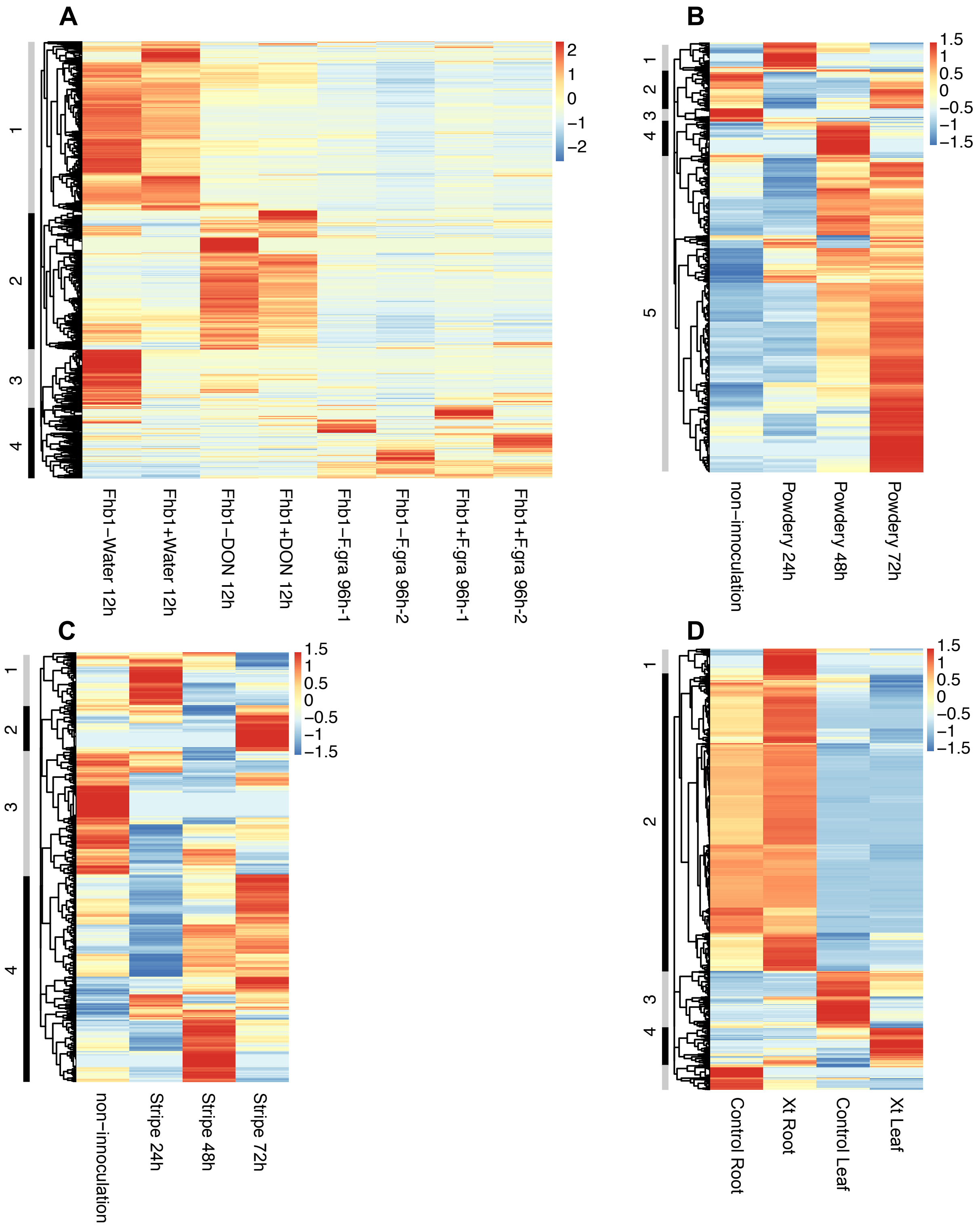


**Figure S8 The expression heatmap of annotated NLR genes from the transcriptome experiment of pathogen inoculation .** Red cell indicated higher expression level, and blue cell represented lower expression level. Left black and grey bar represented the different expression pattern. (A) The expression heatmap from the *Fusarium graminearum* infects wheat spikes and causes Fusarium head blight (FHB). Fhb1+ represented resistant line and Fhb1- represented susceptible lines. -1 and -2 represented two replications. (B) The expression heatmap from the powdery mildew pathogen innoculation. (C) The expression heatmap from stripe rust pathogen innoculation. (D) The expression heatmap from Xanthomonas translucens (Xt) pathogen infection. Plants inoculated with water were used as controls. Data was downloaded from http://202.194.139.32/expression/wheat.html. This figure was generated using R package “pheatmap”. Genes without expression data were not showed. The experiment was carried out with three biological replications, and average values of three replications was used to plot the heatmap.
