# Supplementary material for: Dynamic Diversity of NLR Genes in Triticum and Mining of Promising NLR Alleles for Disease Resistance
Source: Curr Issues Mol Biol. 2021 Aug 17;43(2):965–77. doi: 10.3390/cimb43020069 (PMC8929112; doi:10.3390/cimb43020069)
Supplement: Supplementary file 1 [file cimb-43-00069-s001.zip › Figure S9.pdf]

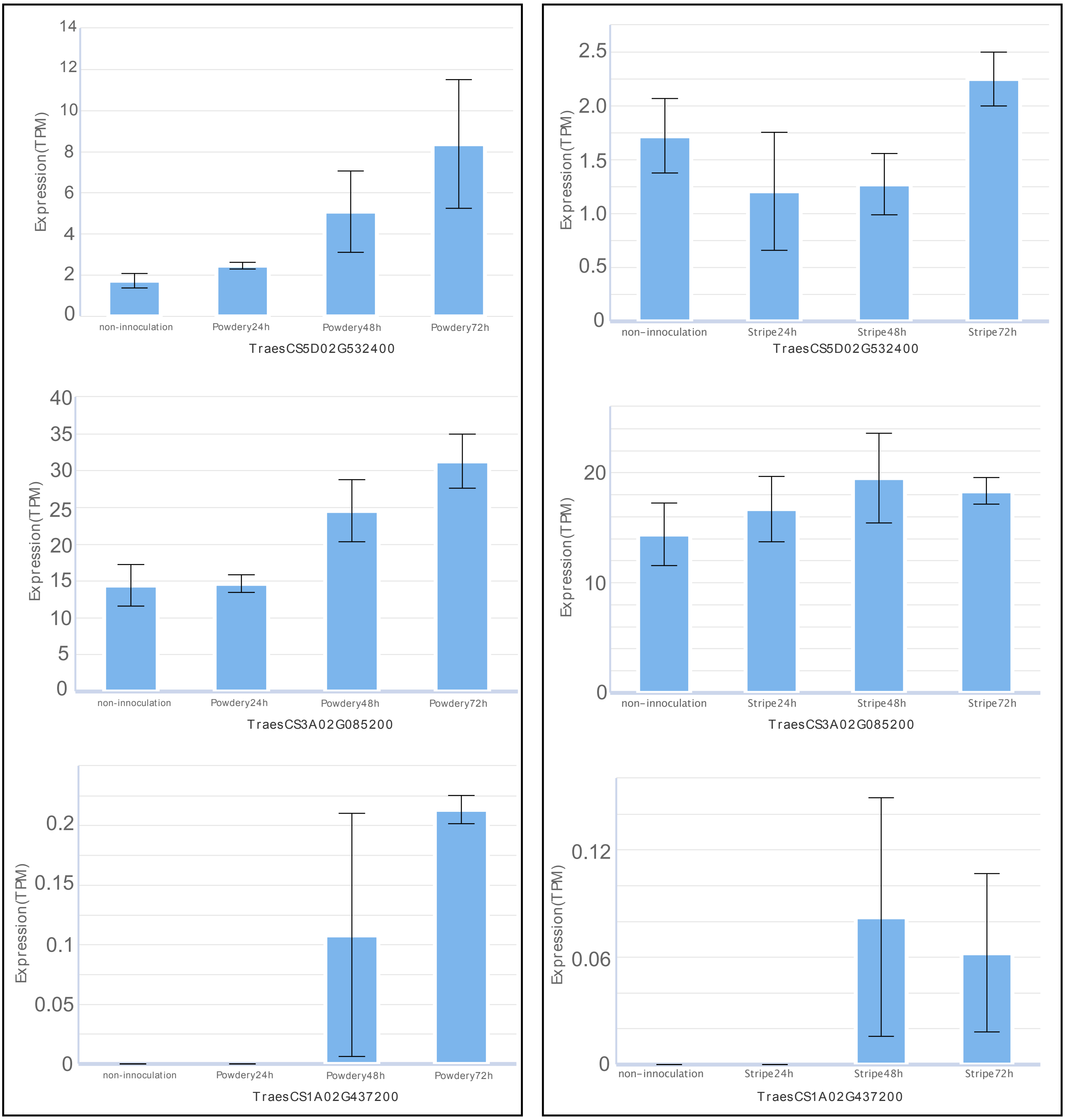

Figure S9 The expression level of three selected genes in different experiment treatments. Left panle showed the expression level of three genes from powdery mildew inoculation experiment, and right panle showed the expression level of three genes from stripe rust inoculation experiment. Data shown are the means ± SD from three replications.
