# Supplementary material for: Dynamic Diversity of NLR Genes in Triticum and Mining of Promising NLR Alleles for Disease Resistance
Source: Curr Issues Mol Biol. 2021 Aug 17;43(2):965–77. doi: 10.3390/cimb43020069 (PMC8929112; doi:10.3390/cimb43020069)
Supplement: Supplementary file 1 [file cimb-43-00069-s001.zip › Figure S1.pdf]

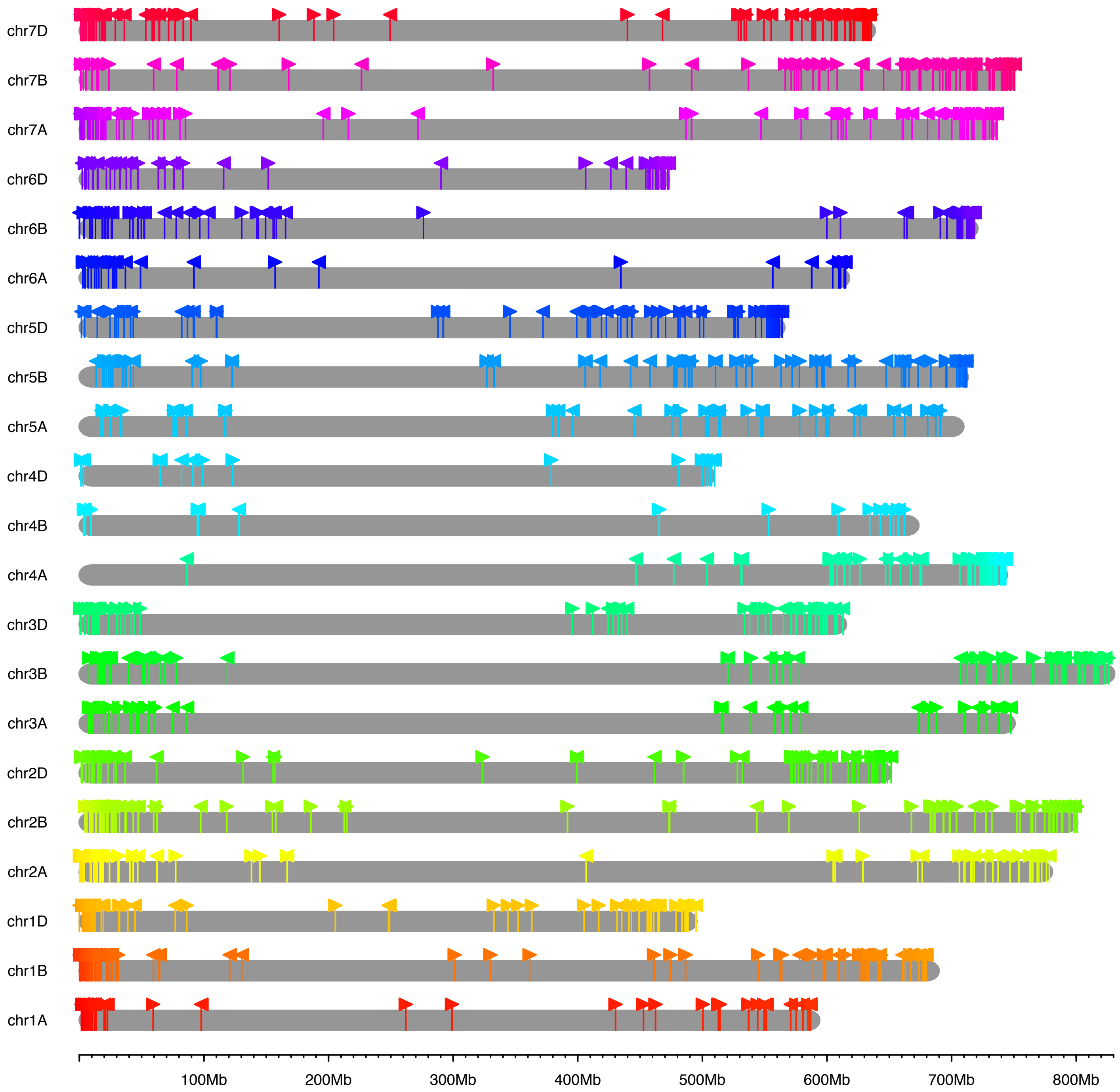

Figure S1 The genomic distribution of identified wheat NLR proteins in 21 chromosomes. Different color lines represented the positions of NLRs in different chromosomes. The arrow of triangle represented the transcription direction. This figure was generated using online website TGT (Triticeae-Gene Tribe, http://wheat.cau.edu.cn/TGT/).
