# Supplementary material for: Dynamic Diversity of NLR Genes in Triticum and Mining of Promising NLR Alleles for Disease Resistance
Source: Curr Issues Mol Biol. 2021 Aug 17;43(2):965–77. doi: 10.3390/cimb43020069 (PMC8929112; doi:10.3390/cimb43020069)
Supplement: Supplementary file 1 [file cimb-43-00069-s001.zip › Figure S2.pdf]

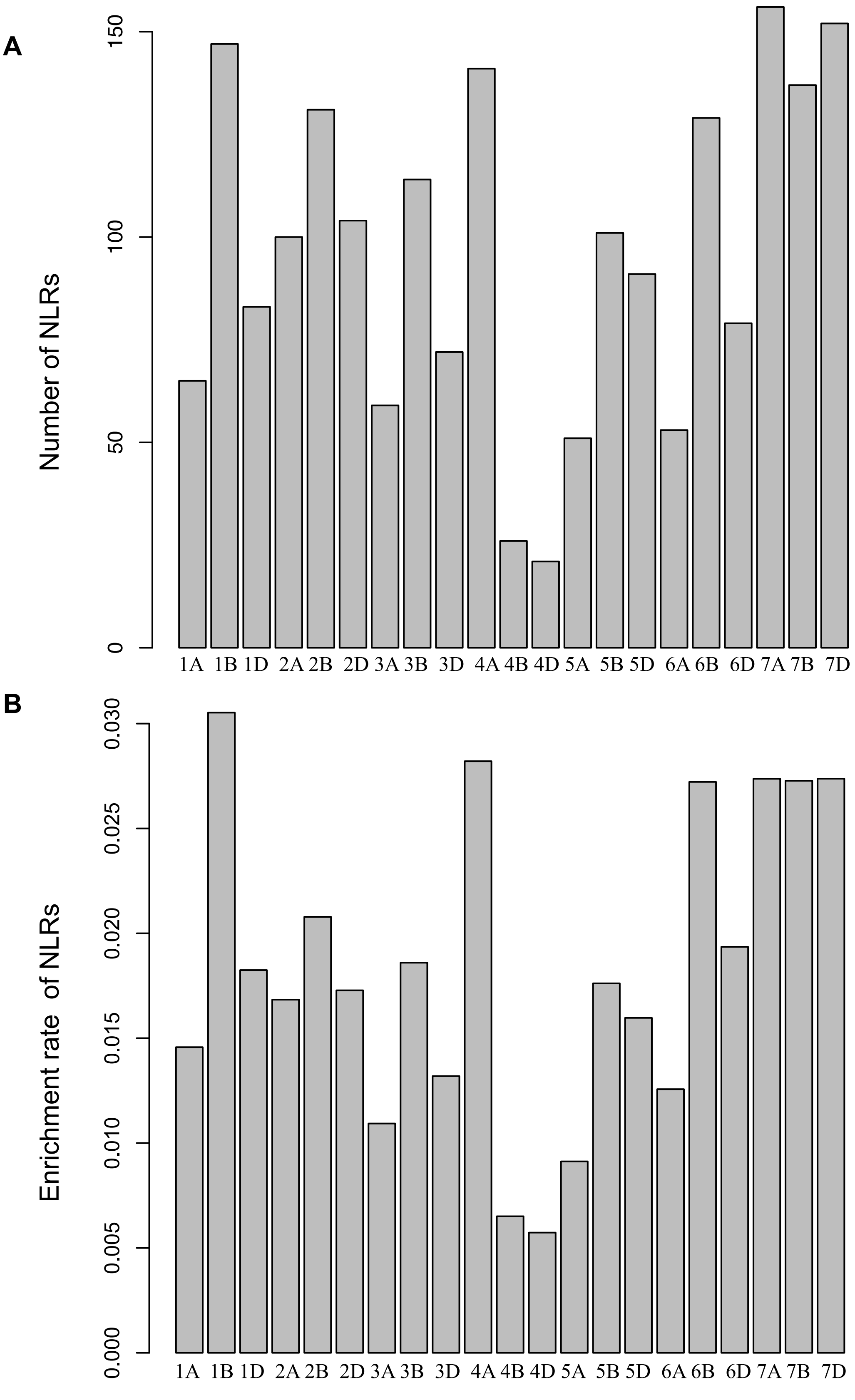

Figure S2 The number of identified NLR proteins in wheat. (A) The number of identified NLR proteins in each of 21 chromosomes.(B) The enrichment rate of identified NLR proteins in each of 21 chromosomes.
