# Supplementary material for: Dynamic Diversity of NLR Genes in Triticum and Mining of Promising NLR Alleles for Disease Resistance
Source: Curr Issues Mol Biol. 2021 Aug 17;43(2):965–77. doi: 10.3390/cimb43020069 (PMC8929112; doi:10.3390/cimb43020069)
Supplement: Supplementary file 1 [file cimb-43-00069-s001.zip › Figure S3.pdf]

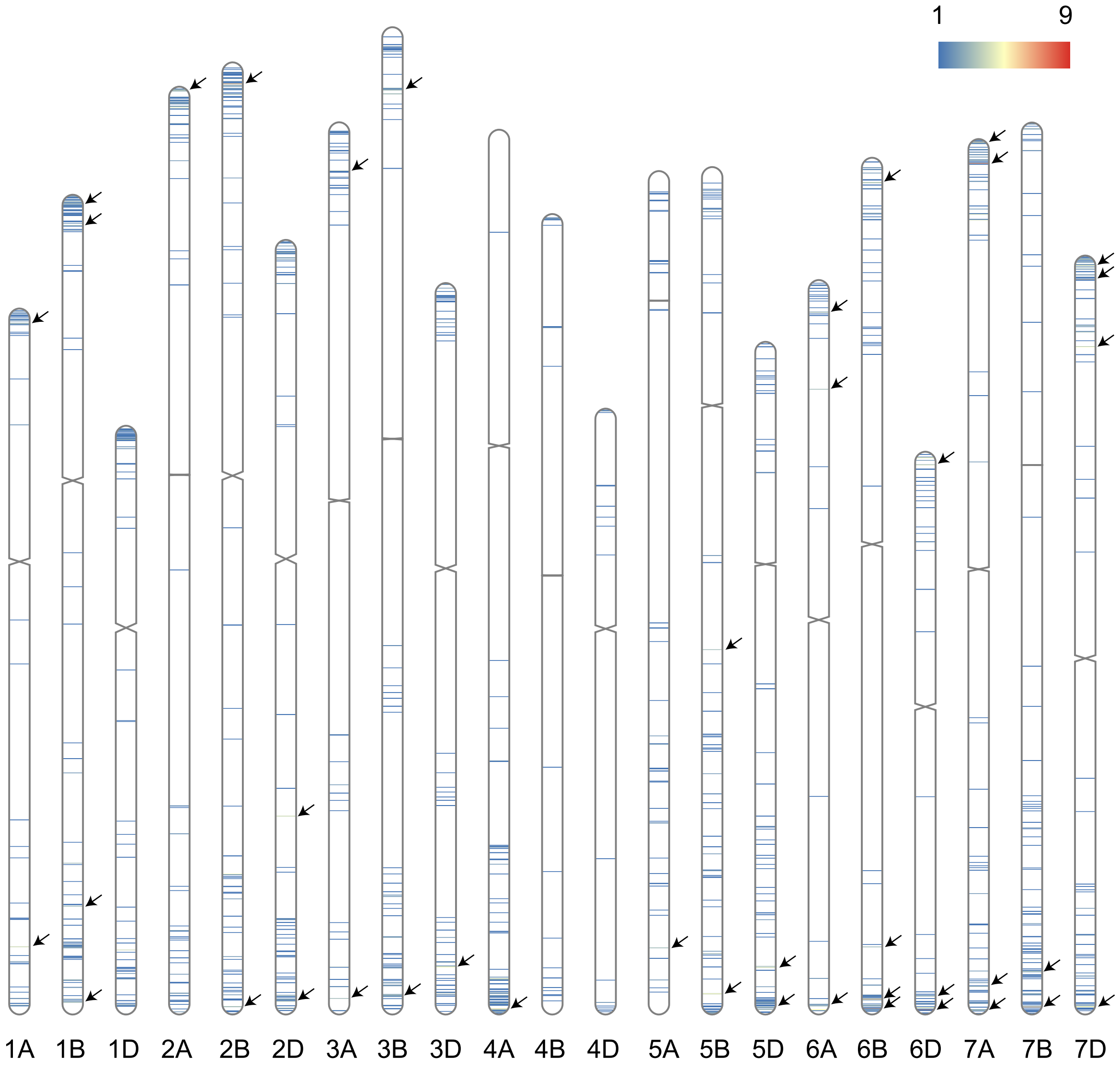

Figure S3 The genomic distribution of 200Kb-windows holding different number of NLR proteins in 21 chromosomes. Different color lines represented number of NLRs in each window. Blue indicated smaller number (such as 1); red indicated larger number (such as 9). Black lines with arrow point to the high-density areas. This figure was generated using R package “RIdeogram”.
