# Supplementary material for: Dynamic Diversity of NLR Genes in Triticum and Mining of Promising NLR Alleles for Disease Resistance
Source: Curr Issues Mol Biol. 2021 Aug 17;43(2):965–77. doi: 10.3390/cimb43020069 (PMC8929112; doi:10.3390/cimb43020069)
Supplement: Supplementary file 1 [file cimb-43-00069-s001.zip › Figure S7.pdf]

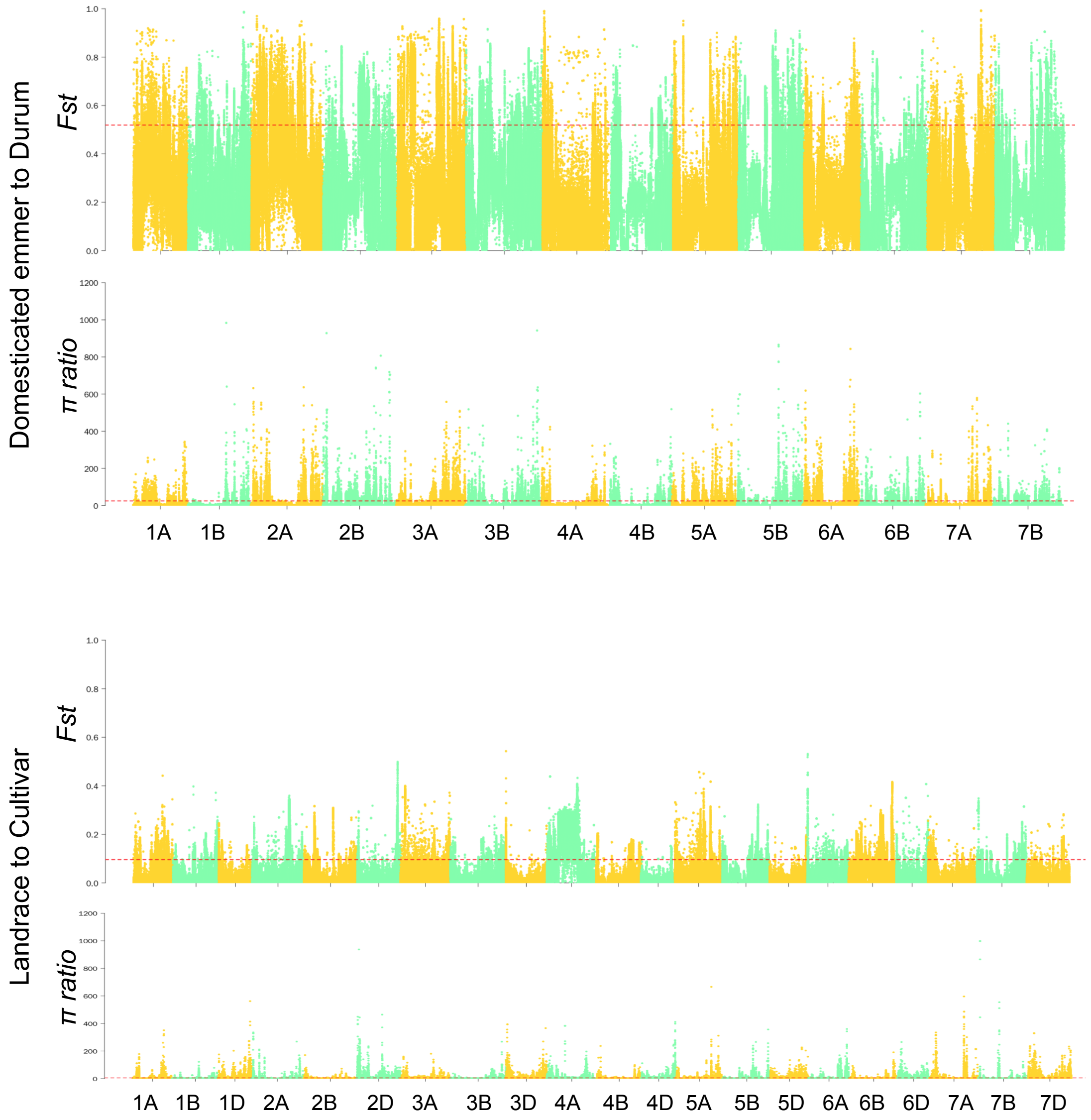

Figure S7 The manhattan plot of Fst values and π (nucleotide diversity) ratio for two improvement processes: Domesticated emmer to Durum, and Landrace to Cultivar. This figure was generated using R package "qqman".
