# Supplementary material for: Dynamic Diversity of NLR Genes in Triticum and Mining of Promising NLR Alleles for Disease Resistance
Source: Curr Issues Mol Biol. 2021 Aug 17;43(2):965–77. doi: 10.3390/cimb43020069 (PMC8929112; doi:10.3390/cimb43020069)
Supplement: Supplementary file 1 [file cimb-43-00069-s001.zip › Figure S6.pdf]

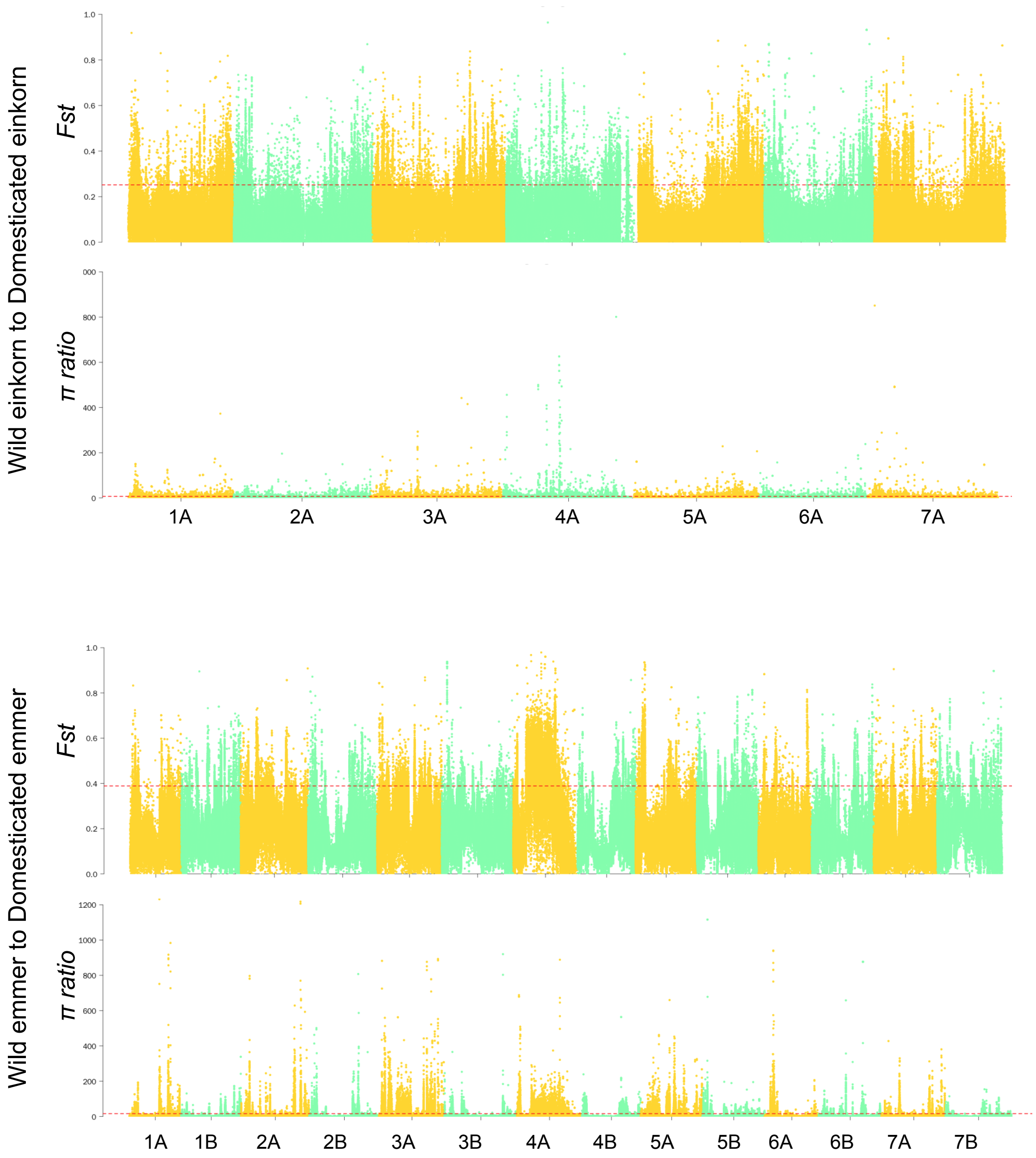

Figure S6 The manhattan plot of Fst values and π (nucleotide diversity) ratio for two domestication processes: Wild einkorn to Domesticated einkorn, and Wild emmer to Domesticated emmer. This figure was generated using R package “qqman”.
