# Supplementary material for: Dynamic Diversity of NLR Genes in Triticum and Mining of Promising NLR Alleles for Disease Resistance
Source: Curr Issues Mol Biol. 2021 Aug 17;43(2):965–77. doi: 10.3390/cimb43020069 (PMC8929112; doi:10.3390/cimb43020069)
Supplement: Supplementary file 1 [file cimb-43-00069-s001.zip › Figure S4.pdf]

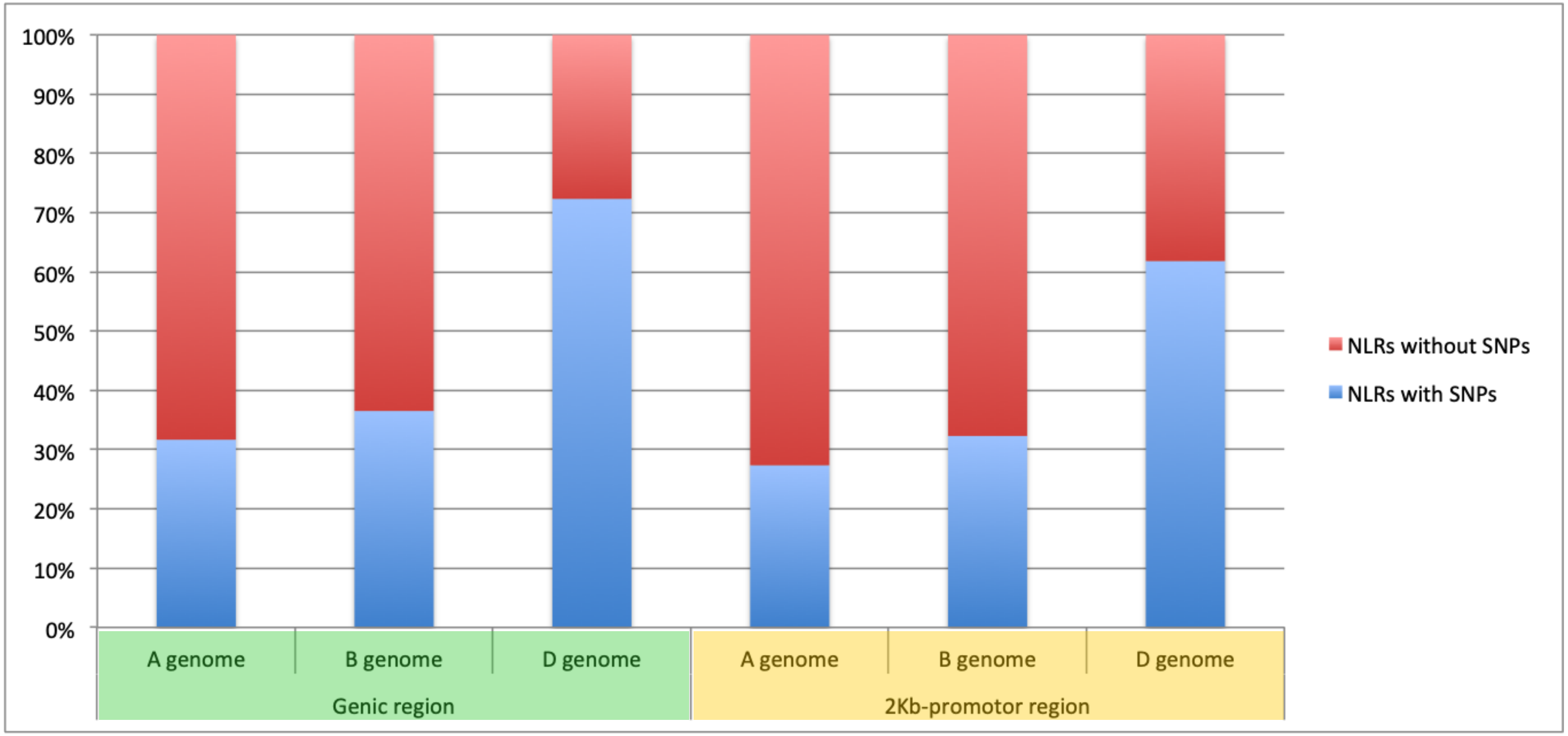

Figure S4 The proportion of NLRs with SNPs and without SNPs in A, B, and D genome lineage. Red bar represents the proportion of NLRs without SNPs, and blue bar represents the proportion of NLRs with SNPs. Left panel (green) is the genic region, and right panel (yellow) is the 2Kb-promoter region.
