# Supplementary material for: Dynamic Diversity of NLR Genes in Triticum and Mining of Promising NLR Alleles for Disease Resistance
Source: Curr Issues Mol Biol. 2021 Aug 17;43(2):965–77. doi: 10.3390/cimb43020069 (PMC8929112; doi:10.3390/cimb43020069)
Supplement: Supplementary file 1 [file cimb-43-00069-s001.zip › Figure S5.pdf]

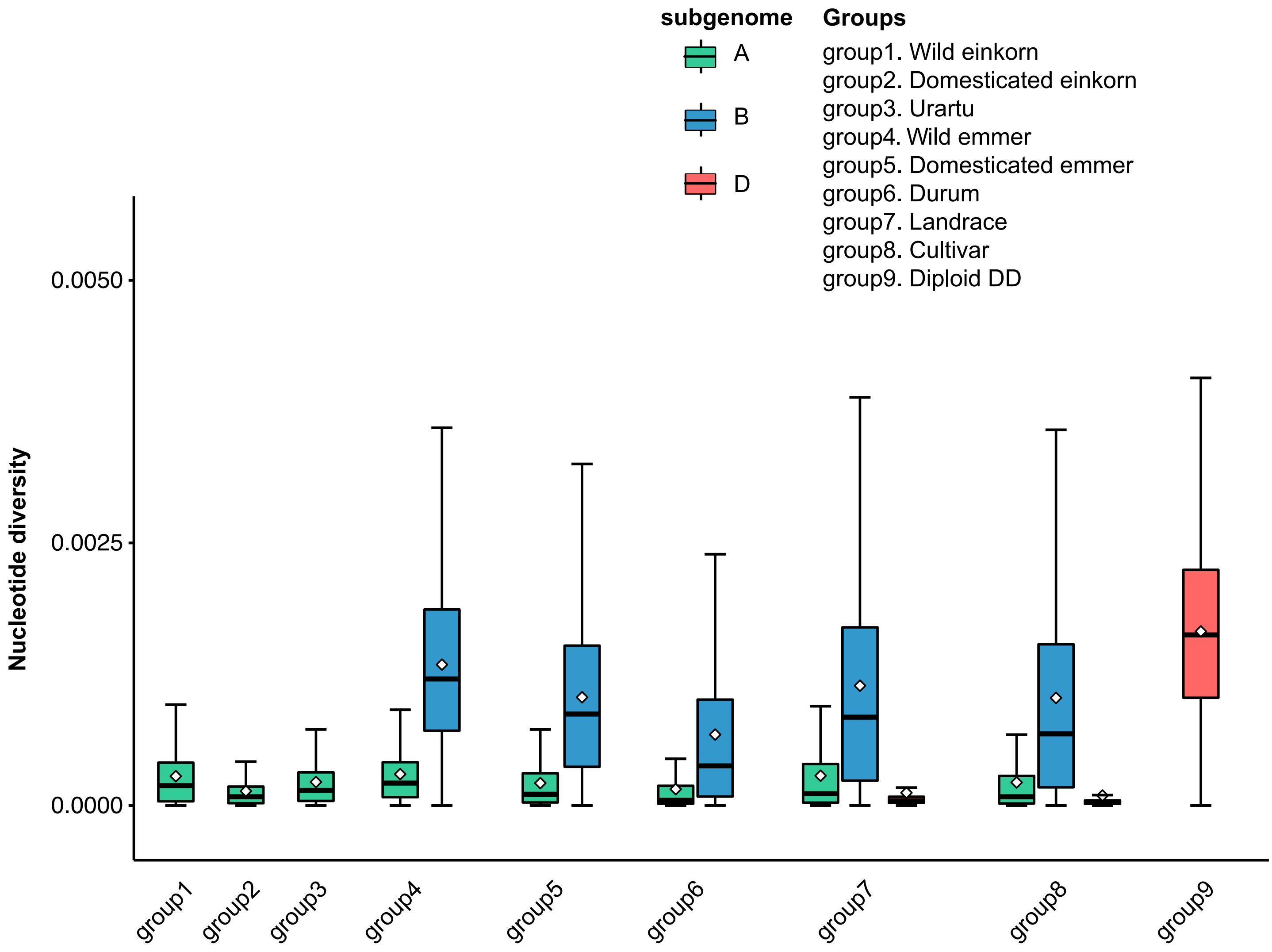

Figure S5 The nucleotide diversity of each of nine groups in different subgenomes. Green box represented A subgenome; blue box represented B subgenome; red box represented D subgenome.
